# Assessment of the Isolated and Combined Impact of β-Glucan and *Lacticaseibacillus rhamnosus* on Cystic Fibrosis Gut Microbiota Using a SHIME^®^ System

**DOI:** 10.3390/nu17233756

**Published:** 2025-11-29

**Authors:** Jazmín Viteri-Echeverría, Joaquim Calvo-Lerma, Jorge García-Hernández, Ana Heredia, Ana Andrés, Andrea Asensio-Grau

**Affiliations:** 1 Instituto Universitario de Ingeniería de Alimentos—Food UPV, Universitat Politècnica de València, Camino de Vera s/n, 46022 València, Spain; jazvi@upv.es (J.V.-E.); anhegu@tal.upv.es (A.H.); aandres@tal.upv.es (A.A.); 2 ALISOST Group, Faculty of Pharmacy and Food Sciences, University of Valencia, Avinguda Vicent Andrés Estellés, 46100 València, Spain; joaquim.calvo@uv.es (J.C.-L.); andrea.asensio@uv.es (A.A.-G.); 3 Advanced Center of Applied Microbiology (CAMA), Universitat Politècnica de València, Camino de Vera s/n, 46022 València, Spain; 4 Departament de Didàctica de les Ciències Experimentals i Socials, University of Valencia, 46022 València, Spain

**Keywords:** beta-glucan, dysbiosis, colonic fermentation, short-chain fatty acids, *Bacillota*/*Bacteroidota*, pediatrics

## Abstract

Background: Cystic fibrosis (CF) is a genetic disorder that disrupts gut microbiota composition, promoting dysbiosis associated with chronic inflammation, impaired nutrient absorption, and poor clinical outcomes. While modulation of the intestinal microbiota through prebiotics, probiotics, and synbiotics has been proposed as a therapeutic strategy, clinical evidence remains limited, especially in children. Objective: This study aimed to evaluate the impact of three supplementation strategies (a prebiotic (β-glucan), a probiotic (*Lacticaseibacillus rhamnosus* GG), and their synbiotic combination) on the gut microbiota and metabolic activity of a CF child faecal donor using a dynamic *in vitro* colonic fermentation model (SHIME^®^). Methods: Microbial composition (16S rRNA gene sequencing), and metabolic activity (quantification of short-chain fatty acids (SCFAs), ammonia, and lactate) were analysed. Results: Results showed that the prebiotic increased alpha diversity; while both the prebiotic and probiotic treatments significantly reduced Bacillota and increased Bacteroidota, modulating the Bacillota/Bacteroidota ratio. The synbiotic treatment showed the most beneficial overall profile, including enhanced production of SCFAs, particularly butyrate and propionate, and increased abundance of *Faecalibacterium* and *Agathobacter*, which are two bacterial genera generally associated with gut health. Notably, the synbiotic also reduced the relative abundance of potentially pathogenic genera such as *Veillonella*, *Megasphaera*, and *Stenotrophomonas*, but paralleled with an increase in *Clostridium* ss 1. Although the probiotic alone showed some positive effects, it was less effective overall compared to the prebiotic and synbiotic approaches. Conclusions: These findings support the potential of synbiotic supplementation as a promising strategy to modulate gut dysbiosis in CF, though *in vivo* studies are needed to confirm the translational relevance of these results.

## 1. Introduction

Cystic fibrosis (CF) is a genetic disorder caused by mutations in the CFTR gene, which result in multiple alterations in the intestinal environment, including thick mucus secretion, reduced intestinal pH, chronic inflammation, and microbial imbalance [1]. These pathological features, together with external factors such as recurrent antibiotics treatments—often required due to repeated respiratory infections—and high-fat dietary intake, contribute to a state of dysbiosis characterised by the overgrowth of pathogenic bacteria in the colonic microbiota [2]. This microbial disruption has been linked to impaired nutrient absorption, suboptimal growth and poorer clinical outcomes in children with CF [3].

Scientific evidence has shown that the gut microbiota in CF is characterised by an increased abundance of inflammation-associated bacterial communities and a reduced richness of immunomodulatory genera [4], overall leading to a specific ecosystem, namely cystic fibrosis-related gut dysbiosis (CFRGD) [5]. Different approaches have been proposed to improve the prognosis and quality of life of individuals with CF, including the modulation of the gut microbiota using biotics [6]. According to the current literature, only a total of 3 studies so far have investigated the efficacy of the use of prebiotics in patients with cystic fibrosis [7,8,9]. In the case of probiotics, more findings have been reported on their impact on respiratory symptoms and, to a lesser extent on the intestinal dysbiosis of the disease, with the most commonly used strains being *Lactobacillus* [10,11]. A recently published systematic review of pre/pro/synbiotics in CF [12] concludes that while some studies have reported positive outcomes, the evidence remains limited, and the results are highly variable. Therefore, more studies are needed to consider these biotic compounds in the nutritional management of CF.

Previous studies have demonstrated the modulation of the microbiota through the administration of β-glucans; Mitsou et al., 2020 [13], indicated that its administration regulates the growth of *Lactobacillus* sp. and *Bifidobacterium* sp., and Joyce et al., 2019 [14], provided evidence that it modulates the composition of the gut microbiota, specifically affecting bacterial taxa involved in the host bile acid metabolism and short-chain fatty acid production, factors that are regulators of host cholesterol homeostasis. Furthermore, a recent study [15] has indicated that *Lacticaseibacillus rhamnosus* GG modulates the intestinal microbiota disorder in short bowel syndrome, in patients with cystic fibrosis-like dysbiosis with low α-diversity, high abundances of Proteobacteria, and low levels of Firmicutes, Actinobacteria, and Bacteroidetes. In a previous study published by our research group, we demonstrated, using a static *in vitro* colonic fermentation model, that the combination of β-glucan and probiotic strains from the *Lactobacillaceae* family may help modulate dysbiosis [8].

Given the importance of evaluating how an altered microbiota metabolises biotic substrates, and to improve data quality and assess the long-term efficacy of such supplementation, a dynamic colonic simulation using the SHIME^®^ *in vitro* model was considered. Dynamic models offer a more accurate representation of *in vivo* intestinal conditions in terms of substrate availability, pH control, transit time, and other physiological parameters [16]. The SHIME^®^ model enables highly reproducible and longitudinal assessments of microbial activity and composition, making it especially suited for hypothesis-generating studies of this nature. In fact, previous research employing SHIME has often begun with single-donor models to establish baseline responses before moving on to multi-donor or cohort-based studies. Our study follows this established approach, aiming to provide an initial, detailed characterisation that can inform and guide future work with larger donor numbers.

Therefore, the aim of the present study was to evaluate the effect of supplementation with a prebiotic (β-glucan), a probiotic (*Lacticaseibacillus rhamnosus*), and their synbiotic combination (β-glucan + *Lacticaseibacillus rhamnosus*) on the gut microbiota composition and metabolic activity of a child with cystic fibrosis, using a dynamic *in vitro* colonic fermentation model.

## 2. Materials and Methods

### 2.1. Design of the Supplementation Treatments: Prebiotic, Probiotic, and Synbiotic

The selection of the type of dietary fibre with prebiotic potential and the probiotic strain was based on findings from a previous study on children with CF, conducted by our research group [8]. A non-starch polysaccharide, β-glucan (Oat; Medium Viscosity) (Main Chain Glycosidic Linkage: β-1,4 and β-1,3) obtained from Megazyme by Neogen^®^ (SKU No. P-BGOM) (Michigan, USA), and a probiotic strain from the *Lactobacillaceae* family, *Lacticaseibacillus rhamnosus* GG (ATCC 53103TM), were selected. The probiotic was cultured in MRS broth (Scharlab, Barcelona, Spain), and the final concentration was adjusted to 10^10^ UFC/mL, using sterile peptone water to preserve it until use (Scharlab, Barcelona, Spain). With these two items, three supplementation treatments were designed as follows: the prebiotic (250 mg/day beta-glucan); probiotic (1.5 mL/day *L. rhamnosus* (concentration of 10^8^ UFC/mL at the colonic compartment)); and synbiotic (250 mg/day beta-glucan and 1.5 mL/day *L. rhamnosus*) (Table 1). These doses were selected based on our previous studies [17,18]. The effects of the three treatments were assessed by simulating supplementation with a dynamic colonic fermentation model on the basal faecal microbiota of a child with cystic fibrosis. 

### 2.2. Obtention of the Faecal Inoculum to Simulate Colonic Fermentation

A stool sample was collected to obtain the faecal inoculum from one donor (n = 1) to simulate colonic fermentation. The sample was obtained from a paediatric patient with cystic fibrosis recruited in the Valencian Community (Spain). The procedure was approved by the Ethics Committee of Universitat Politècnica de València (P03_25-07-2022). The subject was a 12-year-old male with the F508del/G85V genotype, who was not on CFTR modulators therapy and had pancreatic insufficiency (faecal elastase < 15 µg) supplemented with pancreatic enzyme replacement therapy (PERT). The subject presented adequate fat (3.6 g/24 h faecal sample) and protein (0.7 nitrogen g/24 h faecal sample) absorption and anthropometric nutritional status indicators, suggesting adequate weight and growth. The inclusion criterion was a confirmed diagnosis of cystic fibrosis while the exclusion criterion was having taken antibiotics in the last three months. The child’s legal guardians signed the informed consent, and the study was conducted under the regulations of the Declaration of Helsinki.

The decision to work with one faecal donor was based on both scientific and practical considerations. First, our primary objective was to conduct an in-depth, proof-of-concept study using the SHIME^®^ platform to investigate how the cystic fibrosis gut microbiota responds under controlled conditions. Employing a single, well-characterised donor allowed us to minimise inter-individual variability, which is particularly high in CF patients due to differences in genetics, clinical history, and treatment regimens. By controlling this variability, we were able to focus on the mechanistic effects of the intervention without the confounding influence of donor heterogeneity.

### 2.3. Dynamic In Vitro Colonic Fermentation with the SHIME^®^ System

The dynamic gastrointestinal digestion and colonic simulation system SHIME^®^ was employed in this study. The system consisted of six double-jacketed bioreactors, simulating the stomach and small intestine (n = 3) and colon (n = 3). The gastric and intestinal conditions were simulated adjusting pancreatin source (pancreatic enzyme supplements (Kreon^®^ 10000 LU) (Viatris, Canonsburg, PA, USA), intestinal pH (pH = 6), and bile salts concentration (1 mM) to approach the most to the altered conditions found in CF [19,20]. A programmable pump system regulated the flow of digestive contents and the addition of 0.1 M NaOH or 0.1 M HCl to maintain the pH (5.6–5.9). To simulate the *in vivo* colonic environment, the three colonic vessels were inoculated with faecal material from the CF donor. A nutritional medium (PDNM002B, Prodigest^®^) (Ghent, Belgium) was administered in three daily feeding cycles to support the microbial viability.

The experimental design consisted of four consecutive stages. The first was a stabilisation stage lasting 18 days, aimed at allowing the colonic microbiota to adapt to the *in vitro* environment. This was followed by a control stage of 15 days, during which no intervention was applied, to confirm the microbial community’s stability. The third was a treatment stage of 15 days, during which the selected formulations—prebiotic, probiotic, and synbiotic—were administered to evaluate their effects over time. Finally, a post-treatment stage of 10 days was carried out, during which no supplementation was provided, to observe whether any effect persisted after the intervention.

**Figure 1:**
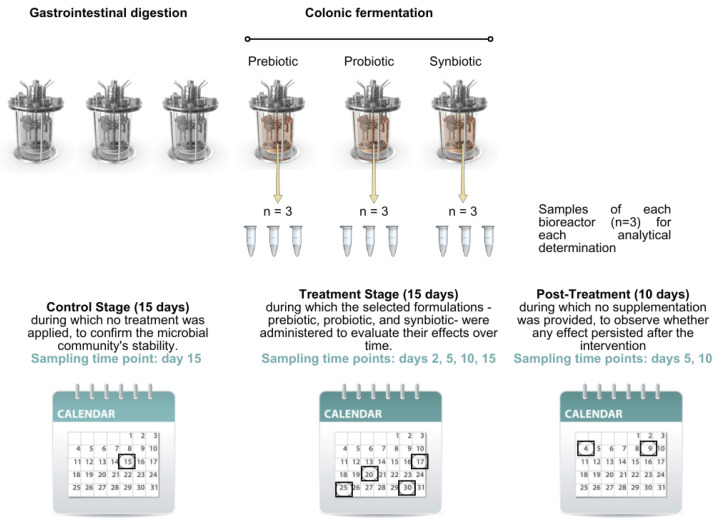
Sampling time points at different stages for analytical determinations. The black boxes frame the day of sampling at each stage.

The digestion process was controlled and parameterised through dedicated software, which regulated volumetric flow rates, the timing of culture medium and pancreatic juice addition, the anaerobic environment, pH, and agitation conditions in the bioreactors. Aliquots were collected from each of the three colonic compartments at specific time points: at the end of the control stage (day 15), along the treatment stage (days 2 [T2], 5 [T5], 10 [T10], and 15 [T15], and mid-term and at the end of the post-treatment stage (days 5 [PT5] and 10 [PT10], respectively) (Figure 1). All samples were immediately stored at −80 °C for subsequent analytical determinations.

### 2.4. Microbiota Composition by 16S rRNA Amplicon Gene Sequencing

To analyse faecal microbiota composition, DNA was extracted from faecal samples using the Stool DNA Isolation Kit (Norgen Biotek Corp^®^ in Thorold, ON, Canada), following the manufacturer’s instructions. The DNA concentration extracted was determined using a Qubit fluorometer (Invitrogen Co., in Carlsbad, CA, USA). Microbiota profiling was performed by amplification of the V3-V4 regions of the 16S SSU rRNA gene using specific primers and Illumina technology. Primer selection followed the guidelines provided [21], and the sequences were in accordance with standard IUPAC nucleotide nomenclature. Primers 515F–806R [22,23] were used following the Illumina amplification libraries protocol. DNA amplicon libraries were generated using a limited cycle PCR (KAPA HiFi HotStart ReadyMix, KK2602) (Roche Diagnostics, Basel, Switzerland), then lllumina sequencing adaptors and dual-index barcodes from Nextera XT index kit v2, FC-131-2001 (Illumina, San Diego, CA, USA) are added to the amplicon in a second PCR. Libraries are then normalised and pooled prior to sequencing. The sample containing indexed amplicons was loaded onto the Nextseq2000 P1 reagent cartridge (reference 20100981, Illumina) and onto the instrument, along with the flow cell. Automated cluster generation and paired-end sequencing with dual indexes reads was performed (2×300bp run) on a MiSeq-Illumina platform at the FISABIO sequencing service in Valencia, Spain. FISABIO sequencing service conducted filtering and quality assessment of raw sequences using the fastp programme [24], which involved removing low-quality nucleotides from the 3′ end (within 10-nucleotide windows with an average quality score under 20) and discarding sequences shorter than 50 base pairs in length. Illumina forward and reverse sequences were joined using the FLASH programme v1 [25]. After that, joined data were processed using the DADA2 package (1.32.0) on R software (R version 4.4.1, released on 14 June 2024). Reads containing undetermined nucleotides and those matching the phiX genome were removed. A denoising algorithm was employed to infer exact ASVs, and chimaeras were removed with default parameters. Taxonomy was assigned to ASVs up to the species level using the SILVA database species train set file (version 138.1).

### 2.5. Metabolite Production: Short-Chain Fatty Acids (SCFAs), Ammonia, and Lactate

Short-chain fatty acid (SCFA) analyses were performed following the protocol described by [26], a SUPELCOWAX™ 10 GC capillary column (30 m × 0.25 mm × 0.25 μm) (Merck, Rahway, NJ, USA) on an Agilent GC7890B-5977B GC-FID gas chromatograph (Santa Clara, CA, USA) equipped with flame ionisation detector FID (Agilent Techonologies, Santa Clara, CA, USA). The oven temperature was initially set at 90 °C for 1 min, then increased to 190 °C at a rate of 5 °C/min, and finally held at 250 °C for 30 min. The column flow rate was 1 mL/min, with helium as the carrier gas. A liquid–liquid extraction was performed as follows: a 2 mL aliquot of samples was transferred into a 15 mL Falcon tube, followed by the addition of 0.5 mL of 9.2 M H_2_SO_4_, a small amount of NaCl, 0.4 mL of internal standard solution (52.9 mM 2-Methylhexanoic acid), and 2 mL of diethylether. The mixture was vortexed for 1 min and centrifuged for 3 min at 3000 rpm, and 550 µL of the organic phase was transferred to a chromatography vial for further analysis. The following SCFAs were quantified: linear (acetic acid (AA), butyric acid (BA), propionic acid (PA), valeric acid (VA)) and branched (BCFAs) (isovaleric acid (IVA), isobutyric acid (IBA)). Results were expressed in terms of millimolar (mM) concentration.

Ammonia and lactate concentration were determined using commercial kits: the Enzytec™ Liquid Ammonia kit (R-BioPharm, Darmstadt, Germany) and the Lactate Assay kit (Sigma-Aldrich, St. Louis, MO, USA), respectively. Both analyses were conducted according to the manufacturers’ instructions. Lactate results were expressed in micromoles per litre (μM) and ammonia results in millimoles per litre (mM).

### 2.6. Statistical Analyses

The microbiota composition and metabolite production were measured in triplicate and reported as mean and standard deviation. Statistical analysis was performed with Statgraphics Centurion XVIII^®^ software (version 19.6.03; Warrenton, VA, USA). A two-way ANOVA followed by Dunnett’s post hoc test was applied to assess statistically significant differences (*p* < 0.05) in metabolic activity and relative microbial abundance at the phylum and genus levels across the different experimental stages: control vs. treatment (after 2, 5, 10 and 15 days of supplementation) and post-treatment (after 5 and 10 days). Relative abundance at genus taxonomic level data were transformed and expressed as increases and decreases with respect to the control values in each treatment. Two-way ANOVA analyses were also performed on a selection of key variables to compare the overall effect of the synbiotic supplementation treatment against the prebiotic and the probiotic. All graphs were generated using GraphPad Prism^®^ software (version 8.4.3, Boston, MA, USA). The alpha diversity (Shannon and Chao indexes) was obtained using R software. The beta diversity (Bray–Curtis scale) was obtained using MicrobiomeAnalyst (web platform; accessed September 2025). Differentially abundant genera were identified with LEfSe in MicrobiomeAnalyst (web platform; accessed November 2025). Prior to biomarker discovery, the feature table was kept at genus level and expressed as relative abundances (TSS-scaled percentages). To avoid artefacts from “count” filters on proportional data, minimum count was set to 0; we applied a prevalence filter of 10% (features present in ≥10% of samples) and excluded very rare features by imposing a minimum relative abundance of 0.1%. No variance filter was applied for LEfSe. For the SHIME design, the class variable was Treatment (prebiotic, probiotic, synbiotic); Bioreactor was specified in the Subject (paired) field to account for within-reactor dependency. When stage-specific effects were assessed, analyses were repeated within each Stage (Control, Treatment, Post) or Stage was used as Subclass. LEfSe was run with Kruskal–Wallis α = 0.05, pairwise Wilcoxon α = 0.05, all-against-all strategy, and an LDA effect size threshold of log10 (LDA) ≥ 2.5. Reported biomarkers therefore reflect features that are statistically significant across groups, consistent within groups, and exhibit a large effect size.

## 3. Results and Discussion

### 3.1. Changes in Microbiota Composition During and After Simulating Supplementation with the Prebiotic, Probiotic, and Synbiotic

Changes in microbiota composition accounted by the three different treatments (supplementation with prebiotic, probiotic, or synbiotic) were assessed at different simulation time points and compared to the baseline values corresponding to the end of the control period and just before supplementation. Of note, the baseline microbiota, despite coming from the same faecal inoculum, resulted in varying proportions in the three different colon bioreactors (i.e., one per treatment) at the end of the control period.

**Figure 2:**
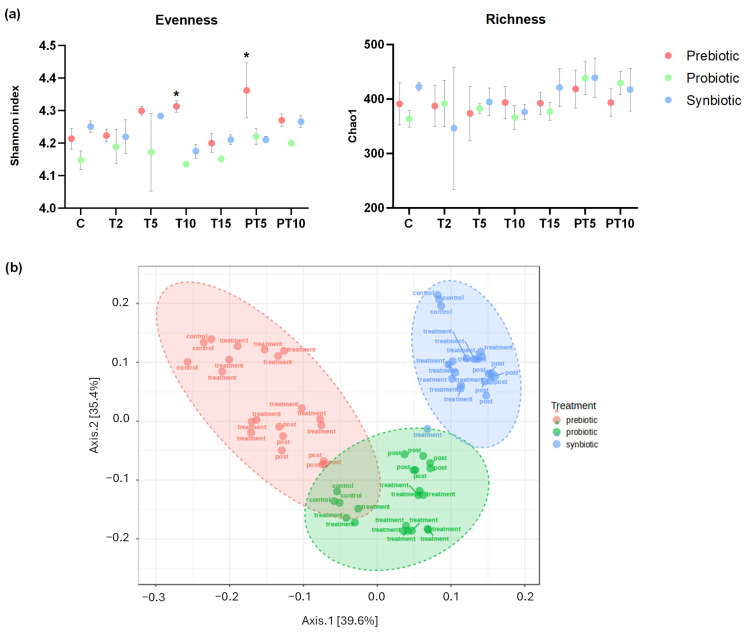
(**a**) Shannon index and Chao1 express the alpha diversity and (**b**) beta diversity (Bray–Curtis) of the faecal microbiota in the colonic environments supplemented with prebiotic (β-glucan), probiotic (*L. rhamnosus*) and synbiotic (β-glucan + *L. rhamnosus*) at six time points of the experiment. (*) Statistically significant differences (ANOVA) of all stages compared to the control at a confidence level of 95% (*p* < 0.05).

In terms of microbial alpha diversity, only the supplementation with the prebiotic (β-glucan) achieved a significant increase as referred to the situation in the control period (Figure 2a). Concretely, supplying the prebiotic increased the evenness (how equal the community is numerically), with statistically different values (*p* < 0.05) of Shannon index after 10 days of treatment and (T10) (4.31) and at 5 days of post-treatment stage (4.36), compared to the control (4.21). In terms of richness (number of different phylotypes present in a community), the supplementation with the prebiotic led to no significant differences over the experiment time points. In terms of beta diversity (Figure 2b), the microbial communities of the different treatments showed statistically significant differences (F-value: 48.019; R-squared: 0.61548; *p*-value: 0.001), with prebiotic supplementation reflecting the greatest change in diversity, while treatment with the synbiotic generated a more consistent and prolonged effect over time. The variations in the microbiota are detailed in greater depth below, comparing relative abundances at the phylum and genus levels.

**Figure 3:**
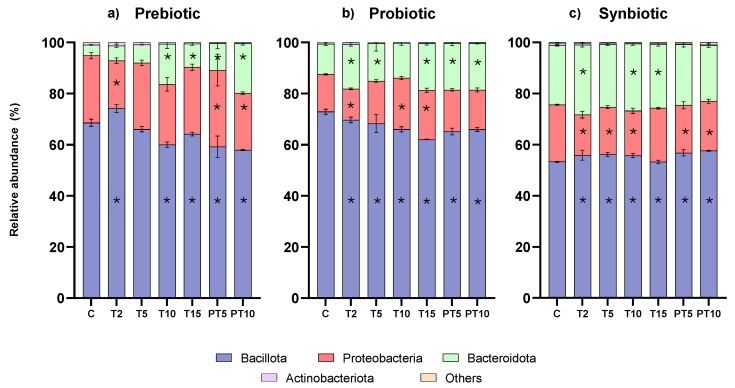
Relative abundance at phylum level of colonic microbiota over the study stages and treatments with (**a**) prebiotic (β-glucan), (**b**) probiotic (*L. rhamnosus*), and (**c**) synbiotic (β-glucan *+ L. rhamnosus*). (*) Statistically significant differences.

Although supplementation with the probiotic and the synbiotic did not significantly improve microbiota diversity, all three treatments induced significant changes in the relative abundance of phyla, as compared to the composition in the control period (Figure 3). At the control stage, the three main phyla were found in the following ranges (three different bioreactors): *Bacillota* (61.20–70.15%), *Bacteroidota* (15.69–22.20%), and Proteobacteria (7.67–18.21%), while *Actinobacteriota* was <1% and *Verrucomicrobiota* was not detected. As expected, the proportion among the three most abundant phyla was altered compared to healthy children. Previous studies have defined those children with CF present with different physiological alterations and specific treatments leading to CFRGD, which is characterised by an unbalanced ratio between *Bacillota*/*Bacteroidota* and also increased Proteobacteria [5,27,28].

Of note, a balanced Bacillota/Bacteroidota ratio has been associated as a marker of gut health [29,30], while keeping Proteobacteria abundance low is also considered as a target of supplementation and dietary strategies to improve gut microbiota [31]. As for the experimental results, the prebiotic and the probiotic supplementation treatments were successful in significantly reducing the abundance of Bacillota while increasing Bacteroidota abundances, helping to balance the ratio (Table S1). Concretely, prebiotic treatment allowed the reduction of Bacillota from 68.6% at the control stage to 64.1% at the end of the treatment (T15) (FDR *p*-value = 0.0043) and to 57.9% at the end of post-treatment (PT10) (FDR *p*-value < 0.0001) (i.e., −10%). Similarly, the probiotic treatment also reduced Bacillota abundance significantly, from initial 72.9% to 62.0% at the end of the treatment period (FDR *p*-value < 0.0001) and to 65.9% at the end of post-treatment (PT10) (FDR *p*-value < 0.0001). In parallel, both treatments enabled the growth of Bacteroidota, from 4.1% (control stage) to 19.4% (PT10) with the prebiotic and from 11.8% (C) to 18.3% (PT10) with the probiotic. However, the synbiotic supplementation treatment showed an inverse effect, leading to a significant statistical increase in Bacillota and a reduction in Bacteroidota, which was not statistically significant. As for the changes in Proteobacteria, fluctuations in relative abundance were registered over the experimental time points with all three treatments. Considering the overall change, the prebiotic and synbiotic treatments accounted for a discrete though significant decrease (i.e., ≈4%) at the end of post-treatment (PT10) (FDR *p*-value < 0.0001).

Going into the details of the genus taxonomic level (Figure S1), a more specific interpretation of the results was enabled. While the bioreactor-specific environment led to slight differences in the microbiota composition found at phylum level, in case of the genera, wider ranges were found, and at the end of the control stage the most abundant genera were *Faecalibacterium* (17.20–46.23%), *Bacteroides* (4.15–23.58%), *Klebsiella* (10.09–18.64%), *Lachnospira* (0.12–19.13%), and *Clostridium* sensu stricto 1 (4.72–6.51%). In fact, statistically significant differences were found between the three bioreactors at this baseline period (Table S2).

**Figure 4:**
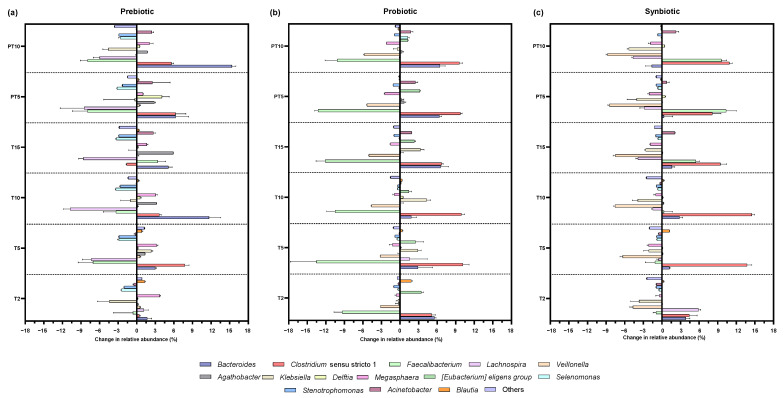
Changes in the relative abundance at genera level in the colonic environments supplemented with (**a**) prebiotic (β-glucan), (**b**) probiotic (*L. rhamnosus*), and (**c**) synbiotic (β-glucan + *L. rhamnosus*) at six time points of the experiment with respect to the control: 2 days of supplementation treatment (T2), 5 days of supplementation treatment (T5), 10 days of supplementation treatment (T10), 15 days of supplementation treatment (T15), 5 days of post-treatment (PT5), and 10 days of post-treatment (PT10).

Changes in genera relative abundance accounted by each supplementation treatment were expressed as increases and decreases to each corresponding control value (Figure 4). More specifically, a significant increase in the *Bacteroides* genus was observed with all treatments, with the prebiotic and probiotic contributing the most to the increase (*p* < 0.0001) at the end of treatment (+5.09%) and (+6.56%), respectively. In contrast, in the post-treatment period the greatest increase (*p* < 0.0001) (+15.36%) was observed with the prebiotic, and a slight but significant decrease (*p* < 0.0001) (−1.63%) with the synbiotic. *Bacteroides* predominantly produce propionate in the colon and have anti-inflammatory properties for the host [32].

As for *Clostridium* sensu stricto 1, there was a significant increase (*p* < 0.0001) in relative abundance after the probiotic (+6.74%) and synbiotic (+9.43%). Of note, *Clostridium* ss 1 comprises a large group of commensals but also pathogenic *Clostridium* species, which in fact has been identified as one of the unusually increased genera in the gut microbiota of cystic fibrosis children [33]. A possible reason for its increased presence relates to the lung–gut axis, as some species of *Clostridium* are among the most resistant pathogens in the respiratory system in this population [2]. Therefore, the increase in *Clostridium* ss 1 could be considered as a negative finding.

In contrast, *Faecalibacterium* was significantly reduced during treatment with the probiotic supplement (*p* < 0.0001) (−12.11%) and after discontinuation (*p* < 0.0001) (−10.18%), unlike the synbiotic treatment, in which the relative abundance increased significantly at T15 (*p* < 0.0001) (+5.47%) and PT10 (*p* < 0.0001) (+9.58%). In the context of cystic fibrosis, it is common to observe a reduced abundance of *Faecalibacterium* [5,27,34]. This genus is a commensal taxon that is considered a marker of intestinal health [35] and is associated with the biosynthesis of SCFAs [28], especially butyric acid [36]. The species *F. prausnitzii* is known for its ability to suppress pro-inflammatory pathways by inhibiting the NF-κB factor and stimulating anti-inflammatory cytokines such as IL-10 and TGF-β [37]. At the same time, butyrate activates G protein-coupled receptors (GPR109A) and increases the expression of tight junction proteins, improving the integrity of the epithelial barrier and reducing mucosal inflammation [38]. Therefore, its increase with synbiotic treatment, as opposed to the effect observed with prebiotic or probiotic treatments, is a remarkable result. So, its increase with the synbiotic treatment, as opposed to the effect with the prebiotic or probiotic treatments, is a noted result.

Another relevant finding was the decrease in *Veillonella*, both during treatment and post-treatment with the probiotic (*p* < 0.0001) (decreases of 5.04 and 5.76%) and the synbiotic (*p* < 0.0001) (decreases of 7.50 and 8.75%). *Veillonella* is a characteristic genus in cystic fibrosis, which dominates the pulmonary microbiota in this population [39,40]. Its commonly increased presence in the colon is linked again with the lung–gut axis, and the mechanisms involved in sputum swallowing [41,42]. The prevalence of some species of *Veillonella* has been associated with increased virulence of other pathogens [43]. However, its role in cystic fibrosis disease is not yet fully understood.

On the other hand, the prebiotic treatment drove the increase (*p* < 0.0001) of *Agathobacter* during the treatment period with an increase of 5.88%, the highest among all the genera. Likewise, the probiotic and the synbiotic induced an increase in this genus in the two stages, but the differences were not statistically significant. Some species within *Agathobacter* are considered as anti-inflammatory microorganisms and are associated with the maintenance of intestinal health [44].

In the case of *Klebsiella*, the prebiotic showed a significant reduction (*p* < 0.0001) only at the post-treatment stage (−4.57%), while the probiotic showed an increase (*p* < 0.0001) (+3.32%) in the treatment. In turn, the synbiotic showed a significant decrease at all stages, in the treatment (*p* < 0.0001) (−2.62%) and post-treatment (*p* < 0.0001) (−5.33%). *Klebsiella* spp., especially the pathogenic species *K. pneumoniae*, and *Megasphaera* spp., are opportunistic pathogens associated with lung infections—though with low evidence, and thus its clinical impact on the disease of cystic fibrosis is still limited [45,46]—and are also found to be specific to the microbiota of children with cystic fibrosis [17]. In this study, the genus *Megasphaera* was significantly reduced (*p* < 0.0001) with the synbiotic along treatment (−1.88%) and post-treatment stages (−1.83%), while the probiotic only achieved a significant reduction (*p* < 0.0001) in the post-treatment stage (−2.20%). A previous SHIME^®^-based study on the effect of *L. plantarum* supplementation also showed a reduction in *Klebsiella* and *Megasphaera* [17]. The opposite occurred with prebiotic supplementation, where a significant increase was evident 10 days after cessation of treatment (*p* = 0.0161) (+2.06%), possibly related to the capability of some *Megasphaera* species to ferment soluble fibres (FOS) [47].

Another result that deserves attention is the reduction of the genus *Stenotrophomonas*, considered as a biomarker of respiratory infections in cystic fibrosis [48], especially the pathogenic species *S. maltophilia, which* has been associated with more recurrent pulmonary exacerbations [49], and its presence in the gut could be related to the gut–lung axis [30]. In this study, the prebiotic β-glucan was able to induce a marked decrease in *Stenotrophomonas* abundance at the treatment stage (*p* = 0.0002) and post-treatment (*p* = 0.0003) with a reduction of 2.88% in both cases. While reductions were also observed with the synbiotic, the change was only statistically significant (*p* = 0.0346) at 15 days of treatment, with a decrease of 0.99%.

### 3.2. Metabolic Activity

**Figure 5:**
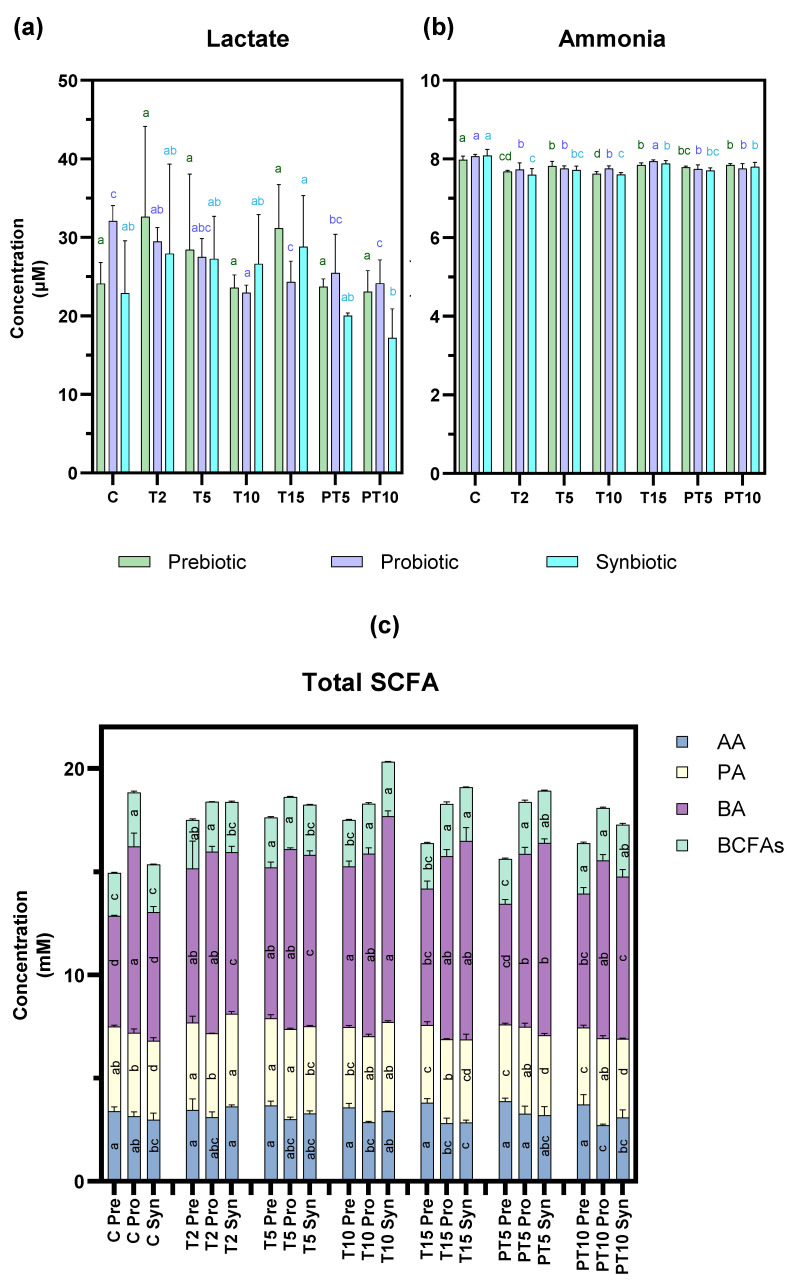
Metabolic activity products of colonic microbiota over the study stages and treatments with prebiotic (β-glucan), probiotic (*L. rhamnosus*), and synbiotic (β-glucan + *L. rhamnosus*). (**a**) lactate, (**b**) ammonia, and (**c**) Total SCFA including acetic acid (AA), butyric acid (BA), propionic acid (PA), and BCFAs, as well as the sum of branched-chain fatty acids, including isobutyric acid and isovaleric acid. a–c: different lowercase letters indicate statistically significant differences between study stages with respect to the control at confidence level of 95% (*p* < 0.05). Letters were assigned in descending order of mean values.

Moving onto the metabolic activity (Figure 5), the following markers were assessed. Lactate and linear-chain SCFAs (acetic, butyric, and propionic acids) as potentially beneficial metabolites, and ammonia and branched-chain SCFAs (isobutyric and isovaleric acids) as potentially harmful metabolites [50]. The concentrations at the different experimental time points were determined and compared to the values of the control stage (baseline values) for all three supplementation treatments (prebiotic, probiotic, and synbiotic).

Focusing first on lactate, the concentration (Figure 5a) was significantly reduced (*p* < 0.05) with *L. rhamnosus* at 15 days of treatment (24.33 µM) and after 10 days of stopping supplementation (24.15 µM) compared to the control stage (32.07 µM), while no significant changes were observed with the other treatments. This result differs from previous studies, where supplementation with the probiotic *L. rhamnosus* significantly increased lactate [8,17]. However, this result is not contradictory as lactate is an intermediate metabolite in the degradation of non-digestible carbohydrates, which in turn is used as substrate by some bacteria to produce SCFAs [51].

Ammonia production (Figure 5b) decreased significantly (*p* < 0.05) with the prebiotic and the synbiotic at the T10 stage and at the post-treatment stage all three treatments continued to reflect a decrease in ammonia concentration. Despite this reduction being statistically significant, it may not be biologically relevant, as other authors have suggested that reductions > 20–30% could be considered as beneficial [52]. The slight reduction could be expected due to the absence of proteins in the culture medium paralleled with the presence of fermentable carbohydrates in the treatments with the prebiotic and synbiotic (beta-glucan) that could have led to the preference for this type of substrate [53].

Similarly to production of lc-SCFAs (Figure 5c), the concentration of acetic acid was only affected by the probiotic treatment, which caused a significant decrease in this metabolite in the post-treatment stage (3.15 mM to 2.71 mM). In contrast, butyric acid production changed significantly with the prebiotic and synbiotic, which significantly increased the control values from 5.35 to 6.24 mM to 6.61–9.62 mM at 15 days of treatment and the increase was maintained to 6.48–7.86 mM at 10 days post-treatment. This finding suggests that the synbiotic combination in this CF context could stimulate cross-fermentation pathways, certain colon bacteria degrading β-glucan into oligosaccharides and monosaccharides, which are then fermented mainly into lactate and/or acetate [54]. In turn, supplementation with the probiotic *L. rhamnosus* contributes to increased lactate production [55]. These metabolites (lactate/acetate) can act as a substrate that other bacterial strains can use for butyrate production via the butyryl-CoA pathway [56]. Regarding propionic acid concentration, a slight but significant decrease was observed with the prebiotic at both stages: treatment (T15) (3.76 mM) and post-treatment (PT10) (3.73 mM) compared to the control value (4.10 mM). This metabolic shift in all the treatments is considered as a relevant finding of the study, as lc-SCFAs serve several metabolic functions associated with colonic health [57].

Finally, bc-SCFA production was influenced by two treatments: the prebiotic significantly increased the concentration of the control from 2.09 to 2.43 mM 10 days after cessation of treatment and the synbiotic showed significant increases at the T15 (2.60 mM) and PT10 (2.52 mM) versus C (2.30 mM) stage. bc-SCFAs result from the degradation of amino acids in the colon and their physiological effects on health are negative, although more evidence is needed to understand their role in cystic fibrosis [58].

Overall, although decreases in certain lc-SCFAs and significant increases in bc-SCFAs were observed in our study, the levels achieved may not be biologically relevant.

### 3.3. Comparison of the Effects of the 3 Supplementation Treatments

In order to provide a practical scope to this study, key study outcomes were selected and compared between the supplementation treatments: synbiotic vs. prebiotic and synbiotic vs. probiotic, to have an objective criterium to answer to the question posed in the introduction (Table 2). According to the FDR-corrected *p*-values resulting from the two-way ANOVA analysis, the prebiotic treatment seemed the most suitable for reducing Bacillota and increasing Bacteroidota. However, it was the synbiotic treatment that exhibited the most positive outcomes, including the highest reduction in Proteobacteria, the highest increase in lc-SCFAs, a not so high increase in bc-SCFAs, an increase in *Faecalibacterium*, and the highest decrease in the CFRGD characteristic genera *Veillonella* and also (along with the prebiotic treatment) in *Megasphaera*. In contrast, the probiotic treatment seemed to be the least beneficial to improve CFRGD, as it accounted for an increase in Proteobacteria, a reduction in lc-SCFAs, and a significant reduction in *Faecalibacterium* (compared to the other two treatments).

**Figure 6:**
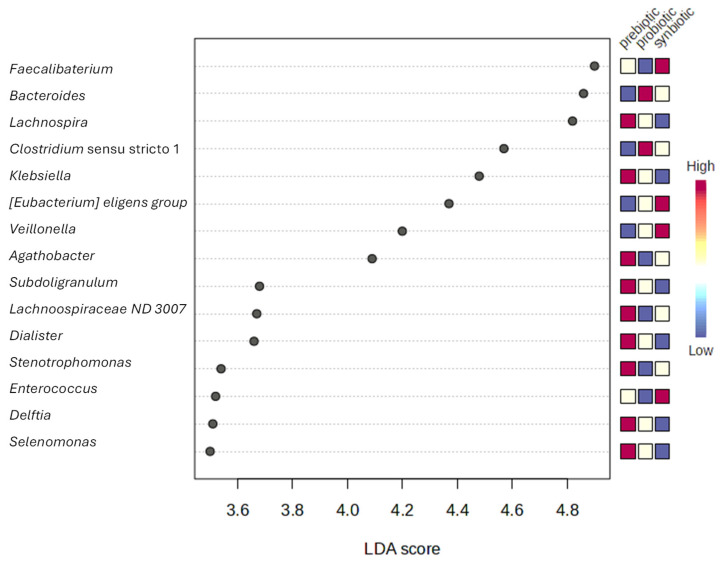
Differentially abundant genera (LEfSe analysis).

Furthermore, to avoid the assessment of individual outcomes and strengthen the conclusions regarding microbial and metabolic shifts mentioned above, LEfSe analysis was performed. This analysis identified 41 discriminatory genera (Figure 6). The highest effect sizes were observed for *Faecalibacterium* (enriched under the synbiotic condition), followed by *Bacteroides* (enriched with the probiotic treatment) and *Lachnospira* (enriched with the prebiotic). Several butyrate-producing taxa (e.g., *Faecalibacterium*, *Eubacterium eligens*, *Agathobacter*) were more abundant in synbiotic samples, whereas other taxa (e.g., *Clostridium* sensu stricto 1, *Klebsiella*, *Lachnospiraceae*) were associated with the alternative treatments. These results highlight biomarkers that are not only statistically significant but also biologically meaningful due to their large effect sizes. For transparency, we also report group medians and log2 fold-changes for significant features to complement LDA scores and facilitate biological interpretation (Table S3).

Before leading to the conclusions, a major limitation of the study has to be acknowledged. While the use of the SHIME^®^ system provided valuable mechanistic information, the use of a single donor limits the direct extrapolation of results to patients with cystic fibrosis, as this approach restricts biological representativeness. However, previous research shows that supplementation with biotics (especially probiotics, the most commonly used in *in vivo* studies) does not produce uniform effects, but rather responses that depend on each individual [59,60,61]. For this reason, in this study a pooled faecal inoculum was not used, with the aim of providing an initial and specific characterisation of the effect of *in vitro* treatments on an individual baseline microbiota that could inform exploratory clinical studies and guide future clinical studies. Furthermore, when working with dynamic models such as SHIME^®^, the number of available bioreactors limits the experimental design. In this experiment, we included several treatments, so it was not technically possible to dedicate independent units to more than one donor at a time [17,18]. On the other hand, some aspects of the simulation were approached in terms of the most translationally relevant and representative conditions in CF, including the simulation of the altered intestinal conditions and the use of supplement-feasible doses of the pre- and probiotic compounds. Finally, our findings should be interpreted as hypothesis-generating and not predictive of clinical efficacy, as only integrated multi-donor and clinical trials can determine the extent to which these microbiota-mediated improvements translate into clinical outcomes.

## 4. Conclusions

This study demonstrates that supplementation with β-glucan, *Lacticaseibacillus rhamnosus*, and their synbiotic combination exerts distinct effects on the gut microbiota and metabolic activity associated with cystic fibrosis-related dysbiosis. While prebiotic supplementation improved microbial diversity and supported butyrogenic taxa, and the probiotic alone achieved modest modulation of bacterial composition, it was the synbiotic strategy that provided the most consistent and beneficial outcomes. Specifically, the synbiotic reduced the abundance of CF-associated pathogenic taxa including *Veillonella*, *Klebsiella*, *Megasphaera*, and *Stenotrophomonas*, promoted the growth of key anti-inflammatory genera such as *Faecalibacterium* and *Agathobacter*, and enhanced the production of short-chain fatty acids, particularly butyrate and propionate. The greater abundance of these beneficial taxa and their metabolic products suggest a functional restoration of SCFA-mediated immunoregulatory pathways.

We consider this design based on the use of the dynamic *in vitro* SHIME^®^ model with a single donor to be a valuable first step, generating new knowledge that broadens our understanding of host–microbiota interactions in cystic fibrosis and lays the foundation for future, more comprehensive *in vivo* research involving integrated assessment of the microbiome and metabolome with controlled trials to evaluate whether restoration of fermentative pathways and reduction in proteolytic metabolites (BCFAs, ammonia) translate into improvements in markers of intestinal inflammation and clinical outcomes (e.g., faecal calprotectin, nutritional status, pulmonary exacerbations).

## Figures and Tables

**Table 1:** Design of the three supplementation treatments assessed in a dynamic colonic fermentation model.

Supplementation Treatment	Beta-Glucan	*L. rhamnosus*
Prebiotic	250 mg/day	-
Probiotic	-	1.5 mL/day
Synbiotic	250 mg/day	1.5 mL/day

**Table 2:** Comparison of the effectiveness of the synbiotic treatment against the probiotic or the prebiotic treatments on a selection of key study outcomes (two-way ANOVA). Information is presented as the mean and standard deviation of the difference between the control (C) and the end of the treatment (T15) values. **Bold values** indicate the most beneficial effects.

	Prebiotic	Probiotic	Synbiotic	Prebiotic vs. Synbiotic	Probiotic vs. Synbiotic
	Mean (SD)			*p*-Value	
Alpha diversity (Shannon index)	−0.014 (0.006)	0.004 (0.03)	−0.041 (0.020)	0.4378	0.1346
Bacillota (%)	−4.44 (1.43)	−10.85 (0.88)	−0.06 (0.70)	0.0023	<0.0001
Bacteroidota (%)	5.04 (0.60)	6.52 (1.30)	1.77 (0.46)	0.0010	<0.0001
Proteobacteria (%)	−0.12 (1.90)	4.61 (0.62)	**−1.27 (0.38)**	0.4075	<0.0001
lc-SCFAs (mM)	1.33 (0.70)	−0.47 (0.76)	**3.44 (0.51)**	0.0142	0.0001
bc-SCFAs (mM)	0.11 (0.07)	**−0.09 (0.20)**	0.30 (0.12)	0.2640	0.0174
*Faecalibacterium* (%)	3.37 (1.28)	−12.11 (1.46)	**5.47 (0.59)**	0.1553	<0.0001
*Clostridium* sensu stricto 1 (%)	**−1.63 (0.08)**	6.74 (0.26)	9.43 (0.96)	<0.0001	0.0001
*Stenotrophomonas* (%)	−2.89 (0.03)	−0.98 (0.04)	−0.99 (0.04)	<0.0001	0.9669
*Veillonella* (%)	0.09 (0.03)	−5.04 (0.40)	**−7.50 (0.38)**	<0.0001	<0.0001
*Megasphaera* (%)	1.61 (0.20)	−1.56 (0.07)	**−1.89 (0.07)**	<0.0001	0.3979

## Data Availability

The original contributions presented in this study are included in the article and Supplementary Materials. Further inquiries can be directed to the corresponding author.

## Supplementary Materials

The following supporting information can be downloaded at https://www.mdpi.com/article/10.3390/nu17233756/s1. Figure S1. Relative abundance at genus level of colonic microbiota over the study stages and treatments with prebiotic (β-glucan), probiotic (*L. rhamnosus*), synbiotic (β-glucan + *L. rhamnosus*). (*) Statistically significant differences; Table S1. Relative abundance at phylum level of colonic microbiota over the study stages and treatments with prebiotic (β-glucan), probiotic (*L. rhamnosus*), synbiotic (β-glucan + *L. rhamnosus*). Mean, SD, and statistically significant differences between study stages with respect to the control at confidence level of 95% (*p* < 0.05) (**Bold values**); Table S2. Relative abundance at genus level of colonic microbiota over the study stages and treatments with prebiotic (β-glucan), probiotic (*L. rhamnosus*), synbiotic (β-glucan + *L. rhamnosus*). Mean, SD, and statistically significant differences between study stages with respect to the control at confidence level of 95% (*p* < 0.05) (**Bold values**); Table S3. LEfSe analysis of bacterial genera significantly associated with the treatments.
