# Supplementary material for: Assessment of the Isolated and Combined Impact of β-Glucan and Lacticaseibacillus rhamnosus on Cystic Fibrosis Gut Microbiota Using a SHIME® System
Source: Nutrients. 2025 Nov 29;17(23):3756. doi: 10.3390/nu17233756 (PMC12694052; doi:10.3390/nu17233756)
Supplement: Supplementary file 1 [file nutrients-17-03756-s001.zip › Supplementary Materials/Table S1.pdf]

Table S1. Relative abundance at phylum level of colonic microbiota over the study stages and treatments with prebiotic (β-glucan), probiotic (*L. rhamnosus*), synbiotic (β-glucan + *L. rhamnosus*). Mean, SD, and statistically significant differences between study stages with respect to the control at confidence level of 95% ($p < 0.05$) (**Bold values**).

		Bacillota			Proteobacteria			Bacteroidota			Actinobacteriota		
**Treatment**	**Stage**	**Mean**	**SD**	**Adj *p* Value**	**Mean**	**SD**	**Adj *p* Value**	**Mean**	**SD**	**Adj *p* Value**	**Mean**	**SD**	**Adj *p* Value**
**Prebiotic**	**C**	68.58	1.37		26.25	1.19		4.17	0.11		0.98	0.09	
	**T2**	74.12	1.58	**0.0002**	18.73	1.08	**<0.0001**	5.85	0.63	0.60	1.27	0.13	1.00
	**T5**	66.02	1.04	0.1954	25.96	1.07	0.9997	7.11	0.10	0.10	0.89	0.14	>0.9999
	**T10**	60.00	1.06	**<0.0001**	23.65	2.66	0.1828	15.78	1.81	**<0.0001**	0.53	0.04	1.00
	**T15**	64.14	0.72	**0.0043**	26.13	1.23	0.9999	9.21	0.62	**0.00**	0.50	0.03	1.00
	**PT5**	59.23	4.16	**<0.0001**	29.93	6.20	**0.0244**	10.38	2.07	**<0.0001**	0.40	0.11	0.99
	**PT10**	57.95	0.27	**<0.0001**	22.18	0.50	**0.0105**	19.41	0.58	**<0.0001**	0.35	0.05	0.99
**Probiotic**	**C**	72.90	1.00		14.59	0.25		11.87	0.64		0.55	0.11	
	**T2**	69.68	1.10	**0.0006**	12.15	0.34	**0.0140**	17.43	0.74	**<0.0001**	0.62	0.02	0.9999
	**T5**	68.30	3.47	**<0.0001**	16.58	0.54	0.0624	14.73	3.01	**0.00**	0.32	0.12	0.9996
	**T10**	66.02	1.01	**<0.0001**	20.03	0.67	**<0.0001**	13.65	0.84	0.12	0.24	0.02	0.9975
	**T15**	62.05	0.13	**<0.0001**	19.20	0.84	**<0.0001**	18.39	0.73	**<0.0001**	0.28	0.08	0.9981
	**PT5**	65.15	1.31	**<0.0001**	16.26	0.46	0.1531	18.19	0.88	**<0.0001**	0.24	0.01	0.9974
	**PT10**	65.99	0.82	**<0.0001**	15.39	0.80	0.8029	18.26	0.24	**<0.0001**	021	0.07	0.9954
**Synbiotic**	**C**	53.30	0.21		22.27	0.21		23.31	0.36		0.69	0.05	
	**T2**	55.89	1.97	**0.0001**	15.84	1.33	**<0.0001**	27.35	0.87	**<0.0001**	0.62	0.16	0.9998
	**T5**	56.13	0.74	**<0.0001**	18.50	0.54	**<0.0001**	24.66	0.44	0.0905	0.44	0.09	0.9951
	**T10**	55.77	0.89	**0.0002**	17.43	0.98	**<0.0001**	26.29	0.60	**<0.0001**	0.29	0.01	0.9527
	**T15**	53.24	0.69	**0.9999**	21.00	0.32	0.1201	25.09	0.79	**0.0129**	0.26	0.03	0.9372
	**PT5**	56.78	1.20	**<0.0001**	18.61	1.39	**<0.0001**	23.79	1.05	0.9084	0.27	0.04	0.9452
	**PT10**	57.62	0.23	**<0.0001**	19.34	0.74	**<0.0001**	21.90	0.78	0.0688	0.23	0.03	0.9187
