# Supplementary material for: Assessment of the Isolated and Combined Impact of β-Glucan and Lacticaseibacillus rhamnosus on Cystic Fibrosis Gut Microbiota Using a SHIME® System
Source: Nutrients. 2025 Nov 29;17(23):3756. doi: 10.3390/nu17233756 (PMC12694052; doi:10.3390/nu17233756)
Supplement: Supplementary file 1 [file nutrients-17-03756-s001.zip › Supplementary Materials/Table S2.pdf]

Table S2. Relative abundance at genus level of colonic microbiota over the study stages and treatments with prebiotic (β-glucan), probiotic (*L. rhamnosus*), synbiotic (β-glucan + *L. rhamnosus*). Mean, SD, and statistically significant differences between study stages with respect to the control at confidence level of 95% ($p < 0.05$) (**Bold values**).

		Bacteroides			*Clostridium sensu stricto 1*			*Faecalibacterium*			*Veillonella*		
**Treatment**	**Stage**	**Mean**	**SD**	**Adj *p* Value**	**Mean**	**SD**	**Adj *p* Value**	**Mean**	**SD**	**Adj *p* Value**	**Mean**	**SD**	**Adj *p* Value**
**Prebiotic**	**C**	4.15	0.10		4.72	0.16		30.66	1.76		0.20	0.04	
	**T2**	5.83	0.64	0.0736	5.13	0.18	0.9805	30.04	1.46	0.88	0.73	0.01	0.9361
	**T5**	7.13	0.11	**0.0001**	12.48	0.58	**<0.0001**	23.61	1.04	**<0.0001**	0.74	0.03	0.9323
	**T10**	15.82	1.81	**<0.0001**	8.37	0.15	**<0.0001**	27.31	0.39	**<0.0001**	0.35	0.02	0.9997
	**T15**	9.25	0.63	**<0.0001**	3.10	0.17	0.0887	34.03	0.53	**<0.0001**	0.29	0.04	0.9998
	**PT5**	10.44	2.09	**<0.0001**	10.99	1.51	**<0.0001**	22.71	1.75	**<0.0001**	0.61	0.13	0.9810
	**PT10**	19.51	0.58	**<0.0001**	10.31	0.47	**<0.0001**	22.73	0.60	**<0.0001**	0.15	0.02	>0.9999
**Probiotic**	**C**	11.92	0.64		5.51	0.13		46.23	1.34		7.51	0.24	
	**T2**	17.50	0.76	**<0.0001**	10.63	0.53	**<0.0001**	36.88	0.43	**<0.0001**	4.36	0.27	**<0.0001**
	**T5**	14.81	3.00	**<0.0001**	15.68	0.87	**<0.0001**	32.71	5.06	**<0.0001**	4.30	0.07	**<0.0001**
	**T10**	13.72	0.84	**0.0174**	15.43	0.63	**<0.0001**	35.74	0.81	**<0.0001**	2.88	0.18	**<0.0001**
	**T15**	18.48	0.72	**<0.0001**	12.25	0.16	**<0.0001**	34.12	0.43	**<0.0001**	2.47	0.18	**<0.0001**
	**PT5**	18.28	0.90	**<0.0001**	15.29	0.22	**<0.0001**	32.97	1.43	**<0.0001**	2.15	0.04	**<0.0001**
	**PT10**	18.36	0.25	**<0.0001**	15.10	0.52	**<0.0001**	36.05	0.72	**<0.0001**	1.75	0.04	**<0.0001**
**Synbiotic**	**C**	23.58	0.36		6.51	0.34		17.20	0.93		9.43	0.29	
	**T2**	27.42	0.90	**<0.0001**	10.91	0.98	**<0.0001**	16.26	1.33	**0.0462**	4.75	0.39	**<0.0001**
	**T5**	24.79	0.46	**0.0056**	20.24	0.76	**<0.0001**	16.08	0.72	**0.0113**	3.09	0.21	**<0.0001**
	**T10**	26.42	0.59	**<0.0001**	21.02	0.21	**<0.0001**	17.52	0.52	0.8882	1.86	0.36	**<0.0001**
	**T15**	25.19	0.75	**<0.0001**	15.94	0.63	**<0.0001**	22.67	0.36	**<0.0001**	1.93	0.24	**<0.0001**
	**PT5**	23.91	1.05	0.8767	14.64	1.08	**<0.0001**	27.55	1.12	**<0.0001**	0.96	0.02	**<0.0001**
	**PT10**	21.96	0.77	**<0.0001**	17.38	0.79	**<0.0001**	26.78	0.41	**<0.0001**	0.67	0.05	**<0.0001**
		Agathobacter			*Klebsiella*			*Megasphaera*			*Stenotrophomonas*		
**Prebiotic**	**C**	0.44	0.06		18.64	0.99		0.75	0.07		3.52	0.06	
	**T2**	0.65	0.01	0.9995	14.22	0.94	**<0.0001**	4.48	0.13	**<0.0001**	1.50	0.04	**0.0199**
	**T5**	1.77	0.14	0.2243	20.98	1.01	**0.0045**	3.95	0.29	**<0.0001**	0.65	0.05	**0.0003**
	**T10**	3.61	0.17	**<0.0001**	17.62	2.57	0.4819	3.82	0.30	**<0.0001**	0.87	0.06	**0.0009**
	**T15**	6.32	0.29	**<0.0001**	18.62	0.98	>0.9999	2.36	0.25	0.0941	0.63	0.05	**0.0002**
	**PT5**	3.26	0.16	**0.0004**	18.32	4.38	0.9943	1.77	0.12	0.4833	1.22	0.02	**0.0056**
	**PT10**	2.14	0.10	0.0667	14.07	0.14	**<0.0001**	2.82	0.57	**0.0161**	0.64	0.06	**0.0003**
**Probiotic**	**C**	0.45	0.01		10.09	0.22		3.59	0.10		1.39	0.02	
	**T2**	0.21	0.01	0.9975	9.74	0.44	0.9845	3.01	0.31	0.8480	0.38	0.03	0.3648
	**T5**	0.36	0.03	0.9998	12.96	0.57	**<0.0001**	2.37	0.47	0.1905	0.56	0.01	0.5698
	**T10**	0.56	0.07	0.9997	14.37	0.47	**<0.0001**	2.67	0.16	0.4545	1.03	0.05	0.9808
	**T15**	0.59	0.03	0.9997	13.40	0.78	**<0.0001**	2.03	0.16	0.0531	0.41	0.03	0.3950
	**PT5**	1.12	0.04	0.7532	10.32	0.50	0.9978	1.11	0.03	**0.0004**	0.27	0.05	0.2735
	**PT10**	0.78	0.12	0.9878	9.63	0.51	0.9389	1.39	0.11	**0.0021**	0.47	0.01	0.4656
**Synbiotic**	**C**	0.06	0.04		16.80	0.42		4.12	0.13		1.34	0.01	
	**T2**	0.16	0.04	0.9996	13.12	1.53	**<0.0001**	3.70	0.42	0.7070	0.43	0.07	0.0608
	**T5**	0.21	0.02	0.9964	14.69	0.44	**<0.0001**	1.96	0.12	**<0.0001**	0.51	0.03	0.0998
	**T10**	0.32	0.02	0.9485	12.90	0.85	**<0.0001**	3.11	0.17	**0.0283**	0.46	0.05	0.0713
	**T15**	0.17	0.02	0.9995	14.18	0.27	**<0.0001**	2.24	0.08	**<0.0001**	0.36	0.04	**0.0346**
	**PT5**	0.11	0.01	0.9998	12.73	1.12	**<0.0001**	2.04	0.09	**<0.0001**	0.42	0.05	0.0537
	**PT10**	0.07	0.02	>0.9999	11.47	0.16	**<0.0001**	2.29	0.38	**<0.0001**	0.67	0.03	0.2579
