# Supplementary material for: Assessment of the Isolated and Combined Impact of β-Glucan and Lacticaseibacillus rhamnosus on Cystic Fibrosis Gut Microbiota Using a SHIME® System
Source: Nutrients. 2025 Nov 29;17(23):3756. doi: 10.3390/nu17233756 (PMC12694052; doi:10.3390/nu17233756)
Supplement: Supplementary file 1 [file nutrients-17-03756-s001.zip › Supplementary Materials/Table S3.pdf]

Table S3. LEfSe analysis of bacterial genera significantly associated with the treatments.

Genera	p-values	FDR	Prebiotic	Probiotic	Synbiotic	LDAScore
Monoglobus	9.7751E-13	4.3011E-11	0.83764	1006.0	22.679	2.7
Dialister	2.5415E-12	5.5913E-11	10466.0	4266.6	1416.1	3.66
Lachnospira	2.5462E-11	3.7344E-10	135100.0	67650.0	2774.2	4.82
Agathobacter	7.3238E-11	7.9886E-10	26193.0	1572.5	5833.4	4.09
Bacteroides	9.0779E-11	7.8886E-10	103860.0	248840.0	162040.0	4.86
Enterococcus	4.163E-10	2.7211E-9	50.115	32.423	6667.7	3.52
Faecalibacterium	4.329E-10	2.7211E-9	274910.0	206940.0	365080.0	4.9
Fusobacterium	4.958E-10	2.7269E-9	109.31	3117.7	377.11	3.18
[Eubacterium] eligens group	8.5482E-10	4.1791E-9	95.504	111.05	46670.0	4.37
Subdoligranulum	1.0175E-9	4.477E-9	12529.0	9371.4	2886.1	3.68
Campylobacter	2.3431E-9	9.3726E-9	14.162	891.05	375.84	2.64
Veillonella	3.1049E-9	1.1385E-8	4404.4	32595.0	36410.0	4.2
[Eubacterium] ventriosum group	4.6218E-9	1.5643E-8	424.4	939.18	44.307	2.65
Flavonifractor	1.3538E-8	4.2548E-8	990.59	267.47	704.92	2.56
Klebsiella	3.1955E-8	9.3735E-8	176250.0	137710.0	115390.0	4.48
Fusicatenibacter	2.1729E-7	5.9755E-7	954.79	365.68	602.97	2.47
