# Supplementary figures and images for: Assessment of the Isolated and Combined Impact of β-Glucan and Lacticaseibacillus rhamnosus on Cystic Fibrosis Gut Microbiota Using a SHIME® System
Source: Nutrients. 2025 Nov 29;17(23):3756. doi: 10.3390/nu17233756 (PMC12694052; doi:10.3390/nu17233756)

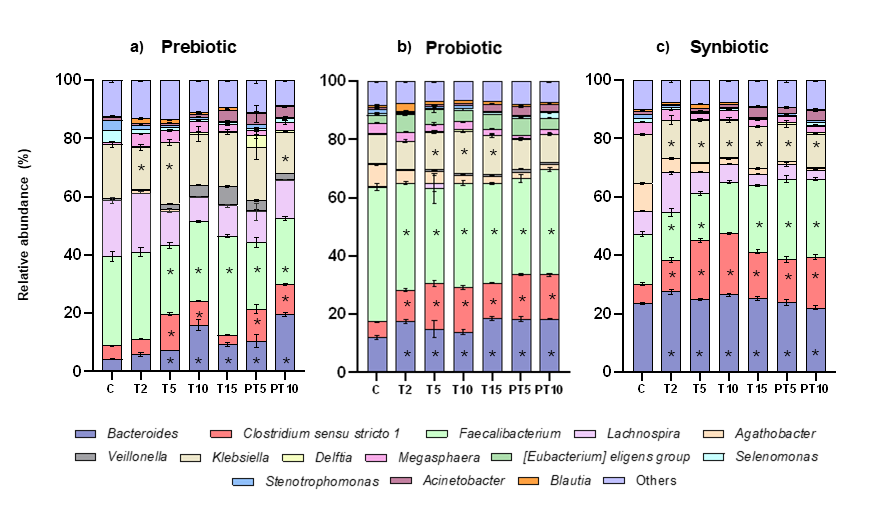

Supplement: Supplementary file 1 [file nutrients-17-03756-s001.zip › Supplementary Materials/Figure S1.png]
